# Postpartum maternal morbidity requiring hospital admission in Lusaka, Zambia – a descriptive study

**DOI:** 10.1186/1471-2393-5-1

**Published:** 2005-02-01

**Authors:** Lisa Vallely, Yusuf Ahmed, Susan F Murray

**Affiliations:** 1 Centre for International Child Health, Institute of Child Health, University College London, London, UK; 2 School of Medicine, University of Zambia, Lusaka, Zambia; 3 King's College London, Florence Nightingale School of Nursing and Midwifery, London, UK

## Abstract

**Background:**

Information on the extent of postpartum maternal morbidity in developing countries is extremely limited. In many settings, data from hospital-based studies is hard to interpret because of the small proportion of women that have access to medical care. However, in those areas with good uptake of health care, the measurement of the type and incidence of complications severe enough to require hospitalisation may provide useful baseline information on the acute and severe morbidity that women experience in the early weeks following childbirth. An analysis of health services data from Lusaka, Zambia, is presented.

**Methods:**

Six-month retrospective review of hospital registers and 4-week cross-sectional study with prospective identification of postpartum admissions.

**Results:**

Both parts of the study identified puerperal sepsis and malaria as, respectively, the leading direct and indirect causes of postpartum morbidity requiring hospital admission. Puerperal sepsis accounted for 34.8% of 365 postpartum admissions in the 6-month period. Malaria and pneumonia together accounted for one-fifth of all postpartum admissions (14.5% & 6% respectively). At least 1.7% of the postpartum population in Lusaka will require hospital-level care for a maternal morbidity.

**Conclusions:**

In developing country urban settings with high public health care usage, meticulous review of hospital registers can provide baseline information on the burden of moderate-to-severe postpartum morbidity.

## Background

*Maternal morbidity *refers to complications that have arisen during the pregnancy, delivery or postpartum period. Every year an estimated 50 million women are affected by maternal morbidity. Defining, interpreting and measuring maternal morbidity, however, is recognised to be difficult and the prevalence of such morbidity (both general and specific) has been poorly described [1,2]. Over the past decade, the nature and extent of postpartum maternal morbidity has received increasing interest in both developed and developing countries with a range of research methods of varying sophistication being used to identify long and short-term and acute and chronic morbidity following childbirth [1,3-8]

The WHO (1998) [1] defines the postpartum period, or puerperium, as beginning one hour after the delivery of the placenta and continuing until 6 weeks (42 days) after the birth of the infant. As the woman recovers from labour, adapts to her new role and reverts physically to her non-pregnant state, it is a special but critical time for both the mother and her infant [9]. Many of the complications leading to postpartum maternal morbidity arise during labour and delivery and in the first 1–2 weeks following delivery; for at least 18 million women these morbidities become long-term and are often debilitating [1]. Major acute obstetric morbidities include haemorrhage, sepsis and pregnancy-related hypertension. Longer-term morbidities include uterine prolapse, vesicovaginal fistulae (VVF), incontinence, dyspareunia and infertility [10].

Fortney & Smith [2] have described 6 dimensions to maternal morbidity: aetiology, severity, duration, time of onset, accumulation and sequelae. However, in many developing countries health services data on postpartum morbidity remains extremely limited. In many settings, data from hospital-based studies is hard to interpret because of the small proportion of women that have access to supervised deliveries and medical care. But, as Fortney and Smith suggest, in areas with good access to and uptake of health care the measurement of the type and incidence of postpartum complications severe enough to require hospitalisation may provide useful baseline information on the acute and severe direct morbidity women experience in the early weeks following childbirth. Zambia's capital city, Lusaka, offers such a context. This paper describes the application of a pragmatic and inexpensive approach to the baseline assessment of the burden of moderate-to-severe morbidity in the postpartum period in this urban African setting, with a view to monitoring trends and identifying preventable risk factors.

## Methods

A network of 23 public sector clinics and a single referral hospital, the University Teaching Hospital (UTH), comprise the public health care system for Lusaka's population of approximately 1.5 million. There is also a small private sector (2% of deliveries) [11] and there are traditional practitioners. An estimated 10.5% of deliveries occur at home. All public clinics provide antenatal care and a postnatal care service at 6 weeks after delivery. In addition, 10 of the clinics provide 24-hour care for labour and delivery and a 1-week postnatal care service. In Lusaka urban district, a recent systematically sampled community survey suggests that there is a relatively high coverage of antenatal, delivery and postnatal services (Table 1). Women interviewees also reported good access to medical treatment for serious problems during pregnancy and in the first month postpartum. Hospital admission data, if used as a proxy for moderate-to-severe morbidity, may therefore be considered pragmatically representative of the population health needs in this setting.

**Table 1:** Uptake of maternal health care services, Lusaka Urban community survey data [11]

**Uptake of maternal health care services**	**% of women reporting (n = 946)**
% of women with 5 or more antenatal check-ups	73%
Proportion of deliveries with a professional attendant	89.5%
% of women attending a postnatal check-up in the 6 weeks postpartum	84%
% of women reporting a "serious problem" in the antenatal or postpartum period who said that they had been able to get medical attention "as soon as they felt they had needed it"	85%

At the University Teaching Hospital, which acts as the district referral hospital for the city, admission and discharge registers are kept in each ward and department. In an earlier retrospective study, analysing referrals for all pregnancy-related complications, undertaken at UTH [12,13], 4% of these cases were identified as referrals in the postpartum period (95/2,892 over a two- month study period). In this study, the hospital registers were used to identify all cases of postpartum morbidity presenting to UTH, and from these to estimate the incidence of, and identify the nature of postpartum morbidity severe enough to require admission for hospital-level treatment.

Data collection was carried out by LV between July and September 2000. Ethical clearance for the study was obtained from the University of Zambia Research Ethics Committee.

For the purpose of this study, the WHO definition of the postpartum period (from delivery until 6 weeks after delivery) was used as the time period inclusion criteria [1]. All women identified as having been admitted to UTH for in-patient treatment for morbidity during this period were included for the purpose of the review, whether or not their morbidity was explicitly "obstetric" in origin. Women who were admitted to hospital to accompany and nurse their babies that had neonatal problems were excluded.

### Retrospective data collection

Relevant admission and discharge registers at UTH were reviewed for the six-month period July-December 1999. Dependent on the type, timing and severity of a postpartum problem, women may be referred or may self-refer to one of three different units within the hospital: (i) Women with early postpartum complications, defined as problems occurring within 24 hours of delivery, are admitted through the labour ward admission room; (ii) Women with postpartum problems occurring more than 24 hours after delivery are referred or may present themselves to the gynaecology filter clinic, from where they may then be referred on to the emergency admission ward; (iii) Cases of breast abscess are generally admitted though the surgical unit. From any of the units women may be then admitted onto a longer stay gynaecology ward for further management and treatment. Women admitted to hospital irrespective of the length of stay were included in the data capture.

Table 2 outlines the identification process and inclusion criteria that were used in each of the wards. The figures are likely to be an underestimate of the total number of postpartum admissions because as is often found in studies of this nature, diagnosis was frequently poorly recorded in the registers. Only women who could be positively identified as postpartum morbidity admissions were included in the final analysis. Any women admitted to medical wards with non-obstetric conditions – such as malaria – in the later part of the puerperium would have been missed. Using admissions registers to identify postpartum cases also excludes any women who were admitted to hospital prior to the puerperium (for example antenatally, or in labour) and subsequently developed postpartum problems requiring prolonged in patient care.

**Table 2:** Identification of postpartum cases and inclusion criteria

	**Emergency Admission Ward**	**Gynaecology Wards**	**Labour Ward**	**Surgical Unit**
**Source of identification of cases**	Admission Register	Discharge Register	Admission Register	Discharge Register
**Inclusion criteria to be defined as postpartum admissions**	All cases recorded as postpartum. All cases with infected caesarean section wound. All cases with infected episiotomy/perineal tears.	All cases recorded as postpartum. All cases with infected caesarean section wound. All cases with infected episiotomy/perineal tears. Any non-obstetric conditions e.g. malaria, PTB, pneumonia, meningitis.	All cases recorded as postpartum.	All cases of breast abscess/mastitis

Cases were crosschecked by name and age between the emergency admission and gynaecology wards and between the short and longer stay surgical wards, to prevent double counting. Cases of breast abscess were also crosschecked between the surgical unit and the gynaecology department to ensure cases had not been referred. Data was entered on Epi. Inf. 6.04 for analysis.

Routine health service statistics for the same six-month study period (July-December 1999) were also collated. There were 19,691 deliveries within UTH and the satellite clinics (5,511 and 14,180 deliveries respectively), of which 1,021 were by caesarean section (an average of 39 per week). Women delivering either at UTH or the clinics were instructed to attend the local satellite clinic for postnatal follow-up visits at week 1 after delivery and again at 6 weeks. Some women were seen at UTH for a postnatal visit at week 1 to follow-up on some complication while those who had a caesarean section were reviewed at 6 weeks. Recorded postnatal check-up at the clinics at 1-week was 40% and 21% at 6 weeks; however, this data was incomplete for some clinics. At UTH, an average of 42 women per week attend the postnatal clinic – most of them after a caesarean section. During the same six-month period, there were 93 maternal deaths at UTH. Of these maternal deaths 8 (8.5%) were known to be due to puerperal sepsis. A much larger number of the women who died had sepsis and stigmata of AIDS (Y Ahmed, personal communication).

### Prospectively identified cases

Due to the limitations of the routine data sources used in the retrospective review, a small cross sectional study was also conducted using prospective identification of cases in order to verify the findings. Over a 4-week period, from 14 August to 10 September 2000, all early postpartum admissions to the maternity unit at UTH were identified through the labour ward admission register. Obstetric case notes were sought and reviewed. Late postpartum cases (>24 hours and up to 6 weeks after delivery) for the same time-period were also identified and recorded by the same means as described for the retrospective study. On the gynaecology ward, cases were identified through daily review of the ward round books, to identify new admissions, and through consultation with the senior ward sister and the ward clerk.

## Results

### Retrospective data

After crosschecking of data to prevent double counting, 365 maternal postpartum admissions to the hospital were positively identified for the 6-month study period July-December 1999. Cases of retained placenta (n = 55), removed in theatre, were not included in the final analysis as it was not possible to differentiate between clinic referrals and UTH deliveries.

#### Referral source and admission status

Of the 365 admissions, 236 (65%) cases were identified through the emergency admission ward, 120 were identified on the gynaecology ward and the remaining 9 cases were identified on the surgical wards. More than half of the emergency admission ward cases were referred from the satellite MCH clinics (Table 3). Two-thirds of all cases were admitted to either the gynaecology transit ward or one of the longer gynaecology wards (97 [41%] and 61 [26%] respectively). A quarter of women were discharged home later the same day after a period of treatment or observation. Six women (2.5%) were admitted to either the special observation unit or medical intensive care unit; five women (2%) were referred to the general adult "filter clinic".

**Table 3:** Sources of postpartum referrals to the emergency admission ward, University Teaching Hospital

**Referral source**	**Number (%) (n = 236)**
Satellite clinics	135 (57%)
Self-referral	28 (12%)
Out-patient department within hospital	12 (5%)
Not documented	61 (26%)

#### Age range

The age range for all cases was 15–48 years. Of all postpartum admissions (365 women), more than half were aged between 20–29 years, a reflection of the fact that the largest number of births takes place within this age group.

#### Nature of morbidities requiring hospital admission

There were 39 different recorded diagnoses across the 365 cases (Table 4). Puerperal sepsis was the most frequent diagnosis, accounting for one-third (34.8%) of all postpartum hospital admissions over the 6-month period. Infection of the reproductive tract, including infected tears and episiotomy, infected caesarean section wound and puerperal sepsis accounted for 47% of all admissions (170/365).

**Table 4:** Identified postpartum admission by diagnosis, University Teaching Hospital, July–December 1999

**Diagnosis**	**Cases (n = 365)**	**Percent of all admissions**
Puerperal sepsis	127	34.8
Malaria	53	14.5
Infected tears/episiotomy	26	7.1
Hypertension	24	6.6
Pneumonia	22	6.0
Infected caesarean section	17	4.7
Anaemia	11	3.0
Breast Abscess	10	2.7
Symphisiotomy	9	2.5
Eclampsia	9	2.5
Puerperal Psychosis	7	1.9
"After pains"	6	1.6
Pulmonary Tuberculosis	5	1.4
Pyrexia (not linked to diagnosis)	5	1.4
Postpartum Haemorrhage	4	1.1
Pre-eclampsia	3	0.8
Retained products of conception	3	0.8
Urinary tract infection	3	0.8
Other (gastroenteritis, meningitis, measles etc)	21	5.8

Malaria was the second most common diagnosis accounting for 14.5% of all cases. Hypertensive disorders including hypertension, pre-eclampsia and eclampsia accounted for 10.9%.

#### Estimating the population burden of moderate-to-severe postpartum morbidity

Using MacKeith et al.'s [14] estimates for public sector coverage of deliveries (87.5%) and the UTH and clinic figures for the period (19,691 deliveries), we estimate that there were approximately 22,000 births in Lusaka during the 6-month period July-December 1999, and 365 hospital admissions for postpartum morbidity were positively identified for the same period. If hospital admission can be taken as a practical proxy measure for the moderate-to-severe end of morbidities, then the burden of moderate-to-severe postpartum morbidity in this urban African population may therefore be estimated to be *at least *1.7% (365/22,000 births). Because this incidence refers only to women admitted live to hospital in the postpartum period, the actual incidence will be somewhat higher, with the addition of postpartum morbidities that occurred in women who were already hospital in-patients, any maternal deaths that occurred outside of the hospital, and any missed late postpartum admissions to medical wards.

### Cross-sectional study of prospectively identified cases

Over the one-month period August-September 2000, 64 admissions to the hospital for postpartum maternal morbidity were identified. The routine hospital procedures differentiate between "early" (up to 24 hours postpartum) and "late" (after 24 hours) postpartum admissions. The former are admitted through the labour ward admissions and the latter through the emergency gynaecology ward. Twenty women (31%) were "early" referrals and 44 (69%) were "late". This categorisation of the data therefore concerns time elapsed between delivery and admission to hospital, and not between time of delivery and onset of the condition.

#### Early-postpartum referrals

Just over one-third (7/20) of the maternal referrals in the first 24 hours after delivery were for retained placenta and just over one-third were for pregnancy- related hypertension or eclampsia (7/20). Three referrals were for postpartum haemorrhage. There were no maternal deaths in this group.

#### Late-postpartum referrals

Referrals from the satellite clinics accounted for 73% of all "late" (>24 hours after delivery) postpartum admissions to UTH; the remaining cases were mainly self-referral. The diagnoses of the 44 cases of late-postpartum admissions in the one-month period are shown in Table 5.

**Table 5:** Late postpartum referrals (Cross-sectional study of prospectively identified cases)

**Diagnosis**	**Number (%) (N = 44)**
Puerperal sepsis	5 (11%)
Malaria	5 (11%)
Pregnancy-related hypertension	5 (11%)
Infected tears/episiotomy	4 (9%)
Infected caesarean section	3 (7%)
Symphisiotomy	3 (7%)
Puerperal psychosis	2 (4.5%)
Secondary postpartum haemorrhage	2 (4.5%)
Meningitis	2 (4.5%)
Retained products	1 (2%)
Eclampsia	1 (2%)
Breast abscess	1 (2%)
Other	10 (23%)

Twenty-one cases (47%) were admitted to the longer stay gynaecology ward. More than half of all cases (52%) were in the 20–29 years age group. Among the 44 women admitted to hospital at more than 24 hours postpartum, 4 died; 2 of these deaths occurred on the longer stay ward. Of the 4 deaths, one could be directly linked to obstetric events (anaemia); the others were related to non-obstetric causes (pneumonia, cryptococcal meningitis, and encephalitis).

## Discussion

This hospital study used time-period based inclusion criteria in its identification of cases of maternal morbidity sufficiently severe to require hospital admission. The majority of morbidity thus identified was directly linked to obstetric causes e.g. puerperal sepsis, infected wounds, and pregnancy-induced hypertension. However, non-obstetric conditions, including malaria and pneumonia were found to have accounted for at least one-fifth of all postpartum admissions in the retrospective review. This mirrors the increased role that indirect causes have been found to be playing in maternal mortality rates in countries such as Zambia [14].

### Data accuracy

We have already outlined some of the practical difficulties with using routine hospital data sources such as admission registers. Admission rates estimated from the small prospective part of the study do not suggest, however, that many cases were lost in the identification process in the larger retrospective part. The former identified an average of 11 late-postpartum maternal admissions to hospital per week in the month observed, and the latter, an average of 14 late-postpartum maternal admissions per week over the six months reviewed. In both parts of the study, puerperal sepsis and malaria were identified as leading causes of postpartum morbidity requiring hospital admission. In the prospective identification of postpartum morbidity requiring hospital admission, puerperal sepsis, malaria and hypertensive disease each accounted for the same number of admission (5 in each) but the numbers are too small to draw any conclusions. Some inaccuracy in the classification of certain morbidities may be expected due to the use of admission diagnoses. It is also a limitation in the design of this element of our study that data collection did not extend to the medical wards. It is therefore not possible for us to ascertain whether, or to what extent, there are medical ward admissions of women in the late puerperium with non-obstetric conditions such as malaria.

### Puerperal sepsis

Puerperal sepsis was the leading cause of postpartum hospital admissions in this population, accounting for 34.8% of all identified postpartum cases in the retrospective part of this study. Other hospital-based studies as well as surveys of women's self-reports of postpartum morbidity report puerperal sepsis as a leading cause of postpartum morbidity in developing countries [5,6,15-18].

Puerperal sepsis cases identified through the retrospective data collection part of the study accounted for more than twice as many cases as the second commonest postpartum morbidity requiring hospital admission, malaria. For the retrospective study the overall rate of puerperal sepsis cases requiring hospital-level care and admission was 0.64% of all supervised deliveries in the public sector services, a rate that falls in between those estimates from earlier hospital-based studies in Niger: 0.22% [5]; and in Nigeria: 1.7% [15]. However, it should again be remembered that in this study, women who delivered in hospital and developed septic complications before discharge would be excluded from this figure. The overall figure can therefore be expected to be higher.

### Other obstetric postpartum morbidity

A number of other obstetric-related postpartum morbidities including anaemia, breast abscess, symphisiotomy, puerperal psychosis, after-pains, urinary tract infection, secondary postpartum haemorrhage and retained products of conception were also identified as late-postpartum referrals through the retrospective study. Anaemia in the postpartum period is not an uncommon health problem [1]. Surveys of women's self-reported morbidity frequently cite symptoms in the postpartum period that could be suggestive of or lead to anaemia, including chronic fatigue [19] and excessive bleeding [15,20,21].

### Non-obstetric postpartum morbidity

Malaria and pneumonia together accounted for one-fifth of all the postpartum hospital admissions that we identified. This finding suggests the usefulness of an approach that employs a "time-period" definition to identify cases rather than simply a set of purely obstetrically-related diagnostic categories.

In all, 14.5% of identified postpartum maternal admissions to hospital were due to malaria. While it is widely recognised that the severity and frequency of malaria is greater in pregnant, compared to non-pregnant women, until recently it was generally thought that the importance of pregnancy-related malaria ends with delivery [22] and malaria is rarely mentioned as an important postpartum morbidity in the obstetric literature. Diagne et al.'s study from Senegal [22], however, was one of the first to suggest that the increased susceptibility to malaria in pregnancy persists up to 60 days after delivery. They found that compared to the non-pregnant state, the incidence of episodes of malaria increased significantly during the second and third trimesters of pregnancy and reached a maximum during the first 60 days after delivery.

A number of factors may modify susceptibility to malaria in the postpartum period. The age at which partial immunity to malaria is acquired is critically dependent on transmission intensity [23,24]. Wide variations are seen in levels of immunity to malaria among Zambian women secondary to geographical and other factors affecting transmission [23]. Many of the malaria cases identified in the study may be the result of recrudescence rather than new infection particularly because the study took place during the transition between dry and wet seasons and study participants were primarily from urban and peri-urban communities. Susceptibility may also be dependent on haematological and nutritional factors as well as HIV status [25].

### The contribution of HIV/AIDS to maternal postpartum morbidity and mortality

Pneumonia and Pulmonary TB were important causes of postnatal morbidity in this study and were likely related to HIV/AIDS. The HIV serostatus was rarely available of the index postpartum cases, though unlinked anonymous testing of HIV in the antenatal population in 4 sentinel sites in Lusaka during 1998 showed a high HIV prevalence of 27.4% [26]. HIV positive women are more prone to postpartum infections including urinary tract infections, chest, episiotomy and caesarean section wound infections [27,28]. Furthermore, in this study, causes of postpartum morbidity included puerperal psychosis, cerebral malaria and HIV related cerebral complications – all of which can be difficult to diagnose with certainty in a malaria and HIV endemic area [29]. Of the 93 cases of maternal mortality during the six-month retrospective study period in 1999, almost a third of the cases were attributed to a presumptive diagnosis of HIV/AIDS and had no other direct or indirect cause of maternal mortality (personal communication, Y Ahmed, 2003).

### Hospital admissions in the first 24 hours following delivery

Review of registers on the labour ward, for both the retrospective and prospective aspects of this study suggest that the majority of referrals in the early-postnatal period (first 24 hours) were for infant rather than maternal indications. Of the early postpartum referrals for maternal indications, retained placenta is the leading reason for referral from clinics to the hospital. This reflects the urban context of the study and the relative ease of transportation that permits a district policy of removal of placenta at hospital level rather than by the practitioner with essential obstetric care skills at the delivery clinic.

## Conclusion

The high public sector maternity care usage in this community permits the low-cost review of routine data to be reasonably meaningful. The caveats are those associated with extraction of data from health facility admission registers, which are not always complete, and which cannot take account of changes in diagnosis or subsequently arising complications. In the absence of more robust data, such reviews, if carried out meticulously, do offer the opportunity to identify the extent of moderate-to-severe postpartum morbidity and the principle causes. They thus may provide the groundwork for detailed condition-specific research to take place exploring aetiology, duration, time of onset and outcome, and the implications for health care provision.

## Competing interests

The author(s) declare that they have no competing interests.

## Authors' contributions

LV: Conceived of the study, conducted the field data collection and data analysis and contributed to the paper.

SFM: Wrote the first draft of the paper, participated in the study design and advised on the data collection and analysis.

YA: Contributed to the study design, advised on the data collection and analysis and contributed to the paper.

All authors read and approved the final manuscript. The views expressed in this article are those of the authors and not of their institutions.

## Pre-publication history

The pre-publication history for this paper can be accessed here:


